# Localisation of clozapine during experimental autoimmune encephalomyelitis and its impact on dopamine and its receptors

**DOI:** 10.1038/s41598-021-82667-6

**Published:** 2021-02-03

**Authors:** Katharina Robichon, Sven Sondhauss, T. William Jordan, Robert A. Keyzers, Bronwen Connor, Anne C. La Flamme

**Affiliations:** 1 School of Biological Sciences, Victoria University of Wellington, P.O. Box 600, Wellington, 6140 New Zealand; 2 Centre for Biodiscovery, Victoria University of Wellington, Wellington, New Zealand; 3 School of Chemical and Physical Sciences, Victoria University of Wellington, Wellington, 6140 New Zealand; 4 Department of Pharmacology and Clinical Pharmacology, Centre for Brain Research, University of Auckland, Auckland, New Zealand; 5 Malaghan Institute of Medical Research, Wellington, New Zealand

**Keywords:** Biochemistry, Diseases of the nervous system, Molecular neuroscience

## Abstract

Multiple sclerosis is a disease characterised by axonal demyelination in the central nervous system (CNS). The atypical antipsychotic drug clozapine attenuates experimental autoimmune encephalomyelitis (EAE), a mouse model used to study multiple sclerosis, but the precise mechanism is unknown and could include both peripheral and CNS–mediated effects. To better understand where clozapine exerts its protective effects, we investigated the tissue distribution and localisation of clozapine using matrix-assisted laser desorption ionization imaging mass spectrometry and liquid chromatography-mass spectrometry. We found that clozapine was detectable in the brain and enriched in specific brain regions (cortex, thalamus and olfactory bulb), but the distribution was not altered by EAE. Furthermore, although not altered in other organs, clozapine levels were significantly elevated in serum during EAE. Because clozapine antagonises dopamine receptors, we analysed dopamine levels in serum and brain as well as dopamine receptor expression on brain-resident and infiltrating immune cells. While neither clozapine nor EAE significantly affected dopamine levels, we observed a significant downregulation of dopamine receptors 1 and 5 and up-regulation of dopamine receptor 2 on microglia and CD4+-infiltrating T cells during EAE. Together these findings provide insight into how neuroinflammation, as modelled by EAE, alters the distribution and downstream effects of clozapine.

## Introduction

Clozapine is an atypical antipsychotic agent used for neurological disorders including schizophrenia and Parkinson’s disease. Clozapine reduces psychosis by antagonising both the serotonin 2A and C (5-HT2A and 5-HT2C) and the dopamine receptor 2 but has also been shown to be immunomodulatory in the central nervous system (CNS)^1, 2^. Given that neuroinflammation occurs during schizophrenia^3^, clozapine’s immunomodulatory ability may contribute to its efficacy in reducing disease severity.

The ability of clozapine to regulate inflammation has been shown to reduce disease in experimental autoimmune encephalomyelitis (EAE), a mouse model useful for the study of neuroinflammatory aspects of multiple sclerosis (MS), a disease characterised by demyelination of the neurons in the CNS^4, 5^. EAE is characterised by the infiltration of autoreactive CD4 + T cells and other immune cells (e.g. monocytes) into the CNS causing an inflammatory state. It has been shown previously that innate and adaptive immune cells are increased in the CNS and that pro-inflammatory cytokines, like interferon gamma and interleukin-17, associated with a high level of inflammation are upregulated^5–7^. We have shown previously that clozapine treatment reduces the expression of pro-inflammatory cytokines and chemokines in the CNS as well as reduction the activation markers (Iba1 and GFAP) on CNS-resident cells^5–7^. However, the precise mechanism by which clozapine ameliorates EAE has not been identified, and because EAE involves not only myelin-specific CD4 + T cells but other peripheral immune and CNS-resident cells, there are many potential targets for clozapine’s protective effects.

A recent phase 1b, randomized, controlled trial in progressive MS patients using low doses of clozapine (i.e. CRISP trial) found that all participants treated with clozapine were withdrawn within 9 days of starting treatment due to adverse events^8^. The CRISP trial results highlight that clozapine’s actions may be altered during MS such that people with progressive MS have an enhanced sensitivity to clozapine. Due to the early withdrawal of all participants in the clozapine group, this trial could not determine whether clozapine was able to impact MS disease as suggested by pre-clinical studies^4, 5, 7^. This surprising finding is at the heart of our rationale for this study, which is to understand why this sensitivity to clozapine occurred (i.e. different pharmacology, metabolism, target expression) so that it can be appropriately used in a therapeutic trial. While EAE does not recapitulate all aspects of MS, it does model CD4 + T cell-driven neuroinflammation^9^. As such, EAE is a useful model to dissect clozapine’s mechanism of action and to understand how neuroinflammation itself may alter clozapine’s effects. Thus, we investigated which tissues are targeted by clozapine by determining its tissue localisation and distribution as well as the expression of its target receptors.

Clozapine antagonises dopamine receptors (DR), which can be separated into two subtypes, D1 subtypes (DR1 and DR5) and D2 subtypes (DR2, DR3 and DR4), based on modulation of cAMP production^10^. All DRs are highly expressed in the brain^10^ and on immune cells^11^, and have shown to be involved in mental disorders and neuroinflammatory diseases like schizophrenia, depression, Parkinson's and Huntington’s disease^12, 13^. Given clozapine’s affinity for DRs, it has been reported to displace dopamine from the receptor within less than one minute^14, 15^, directing it towards other receptors and causing it to have unanticipated effects. Importantly, previous research has shown that selective DR1 antagonism reduces EAE^16^ while selective DR2 antagonism exacerbates EAE^4, 16^ indicating that dopamine receptors are involved in EAE. Thus, we have focused our investigation on dopamine.

Dopamine is a neurotransmitter with many distinct functions including in the reward pathway and motor control. Previous studies using the EAE model showed that increasing dopamine levels improved the symptoms of EAE^17^ while depletion worsened disease^18^. This could be due to the immunomodulatory effects of dopamine on T cells leading to increased production of tumour necrosis factor-α and interleukin-10^19^. However, the context of the tissue and the inflammatory state seems to determine the effect dopamine on a given T cell subset and function^20^. The modulation of the function and phenotype of monocytes and macrophages is another way how dopamine might improve symptom of EAE^21^. Incubation of monocytes and macrophages in vitro with dopamine improved their phagocytic activity. Thus, these studies highlight an important role for dopamine as immunomodulator in EAE and suggest a change in dopamine levels by clozapine might be one means by which clozapine reduces EAE disease severity.

Liquid chromatography-coupled mass spectrometry (LC–MS) is an important tool for drug metabolism and pharmacokinetic studies and is commonly used for metabolite identification, quantification, toxicology and pharmacokinetics due to its high sensitivity, rapid analysis and high specificity^22^. However, LC–MS only measures absolute levels without any information about localisation and distribution. In contrast, matrix-assisted laser desorption ionization (MALDI) imaging mass spectrometry (IMS) analyses tissue sections for the distribution of a range of unlabelled targets^23^, and thus provides spatial information.

In this study, we used MALDI IMS in combination with quantitative LC–MS time of flight analysis to investigate the localisation and distribution of clozapine and dopamine in brain regions and peripheral organs. In addition, we analysed dopamine and DRs in the CNS to better understand clozapine’s actions during EAE-associated neuroinflammation.

## Results

### Clozapine was detected in the brain by MALDI IMS

**Figure 1:**
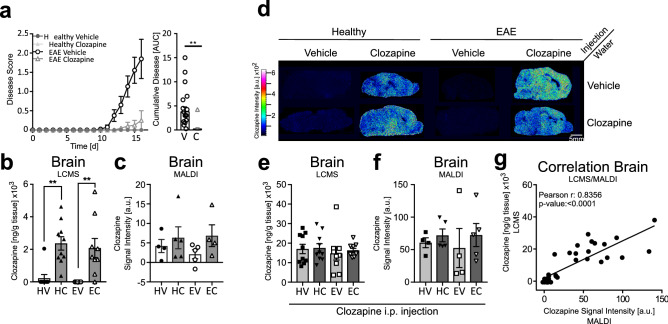
Clozapine in the brain of healthy and EAE mice. Mice were treated with clozapine or vehicle 1 day prior to EAE induction in the drinking water and scored daily (0: normal to 5: moribund). 30 min before euthanasia mice were injected i.p. with 10 mg/kg clozapine **(a)** Disease course of mice and disease burden assessed by area under the curve analysis. Shown are the means and SEM of individual mice from two experiments combined (n = 20 mice/group), Paired t test, two-tailed **p < 0.0021 **(b,d)** Absolute concentration of clozapine in mouse brain measured with LC–MS (n = 10 mice/group) one-way ANOVA repeated measure followed by Tukey Test, **p < 0.01 **(c,e)** Quantification of clozapine intensity by MALDI IMS in the whole mouse brain (n = 4–5 mice/group) **(f)** Positive correlation of absolute clozapine concentration by LC–MS and clozapine intensity by MALDI; Pearson correlation coefficient: 0.8477 with p-value < 0.0001. **(g)** Representative MALDI images from all eight treatment groups.

Clozapine readily passes through the blood–brain barrier to mediate its anti-psychotic effects^24^. Previous studies have shown that clozapine administered orally at a concentration of 60 mg/kg; reduced the onset and the severity of EAE in the mice^5, 25^; therefore, we assessed the distribution and localisation of clozapine in the brains of healthy and EAE mice treated with vehicle or clozapine. As reported previously, clozapine treatment reduced the severity of disease as measured by the disease score and the area under curve of the disease course (Fig. 1a, **p < 0.0021, V-Vehicle, C-Clozapine). Absolute quantification of clozapine levels from brain homogenates detected by LC–MS showed around 2 µg per gram tissue in the brains of healthy and EAE clozapine-treated mice with no difference detected between healthy and EAE mice (m/z 327.137, Fig. 1b, p < 0.01, HV-healthy vehicle, HC-healthy clozapine, EV-EAE vehicle, EC-EAE clozapine). Because we found that clozapine is readily detected by MALDI IMS (Fig. S1c,d), we used MALDI IMS to evaluate clozapine levels in the brain but found that the levels were close to the background (vehicle-treated mice; Fig. 1c, HV-healthy vehicle, HC-healthy clozapine, EV-EAE vehicle, EC-EAE clozapine). These low levels may be due to the rapid clearance of clozapine, which has a half-life of approximately 1.5 h in rodents^26^. Therefore, to increase the detection of clozapine in the mouse brain with MALDI IMS, mice were administered increasing concentrations of clozapine, and the brains were removed 30 min later when clozapine can be detected in all organs by PET scanning^26, 27^. As seen in Fig. S1b,e, all tested doses of clozapine could be detected in the brains of healthy mice and the intensity of clozapine correlated to the dose administered (Fig. S1b,e). However, at the highest concentration of clozapine, sedation, which is a well-known side effect of clozapine, occurred^28^. Thus, a lower dose was selected, with 10 mg/kg clozapine giving reliable results. While there could be potential differences in localisation, concentration and distribution between chronic exposure to clozapine in the drinking water and a single injection of clozapine, this method readily enabled detection and quantification of clozapine in the brain (Fig. 1c,f respectively). Figure 1d shows representative images for all eight treatment groups. No differences were detected by LC–MS or MALDI IMS between healthy and EAE mice, with or without clozapine in the drinking water (Fig. 1e,f HV-healthy vehicle, HC-healthy clozapine, EV-EAE vehicle, EC-EAE clozapine). A Pearson correlation between the intensity of clozapine (MALDI IMS) and the absolute concentration of clozapine (LC–MS) showed a highly significant correlation (Fig. 1g) supporting the quantitative use of MALDI IMS for these studies.

### Clozapine preferentially localised to specific brain regions, but its distribution was not altered by EAE

**Figure 2:**
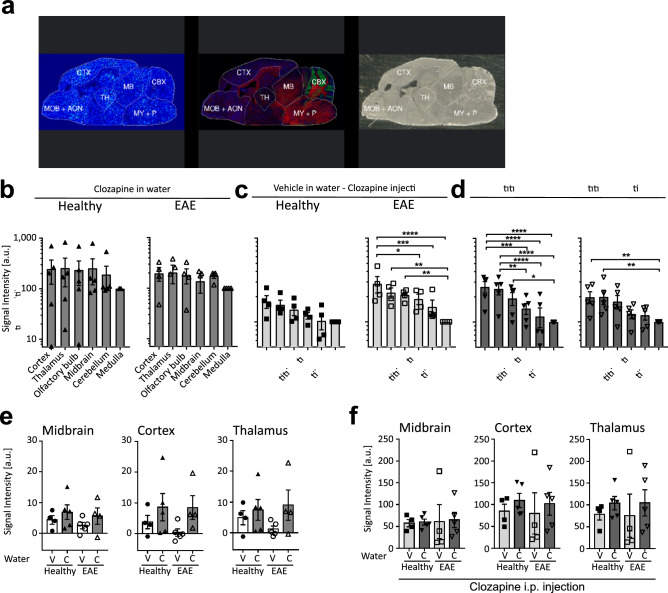
Segmentation of mouse brain into different brain regions and clozapine intensity**. (a)** Optical image, clozapine MALDI image and different lipid distributions for the segmentation into different brain regions. *CBX* cerebellum; *CTX* cortex; *MB* midbrain; *MOB + AON* main and accessory olfactory bulb; *MY + P* medulla and pons; *TH* thalamus. Clozapine intensity in the different brain region measured by MALDI in mice with clozapine in the drinking water only **(b)** and vehicle **(c)** or clozapine **(d)** in the drinking water and i.p. re-injection of 10 mg/kg clozapine. (n = 4–5 mice/group), two-way ANOVA repeated measure followed by Sidak’s multiple comparison test, *p > 0.05; **p > 0.01; ***p > 0.001, ****p > 0.0001 **(e)** Clozapine intensity in midbrain, cortex and thalamus in healthy and EAE mice **(f)** and after clozapine re-injection C – clozapine; V—vehicle.

The MALDI images showed that clozapine was not evenly distributed in the brain. To evaluate regional differences, we segmented the brain into six regions based on the optical images and specific lipid distributions detected by MALDI IMS (Fig. 2a). Analysis of these brain regions (CBX—Cerebellum; CTX—Cortex; MB—Midbrain; MOB + AON—main and accessory olfactory bulb; MY + P—Medulla and Pons; TH—Thalamus) showed high clozapine intensity in the cortex, the thalamus and the olfactory (Fig. 2b,c,d; *p > 0.05; **p > 0.01; ***p > 0.001, ****p > 0.0001). Clozapine intensity was significantly increased in mice pre-treated with clozapine in the drinking water compared to vehicle in healthy mice (Fig. 2c,d) but the localisation to different brain regions did not change. To determine if neuroinflammation altered clozapine distribution, we compared healthy and EAE mice treated with clozapine in drinking water but found no overall significant difference (Fig. 2e, V-Vehicle, C-Clozapine, S1f). Additionally, no differences were observed between healthy and EAE mice if reinjected with clozapine in the presence or absence of clozapine in drinking water (Fig. 2f, V-Vehicle, C-Clozapine, S1g). Taken together, these data suggest that while clozapine seems to localise to specific brain regions, neuroinflammation in EAE and chronic or acute clozapine exposure do not alter its distribution.

### During EAE, clozapine and norclozapine levels were significantly elevated in the serum, but not other peripheral organs.

**Figure 3:**
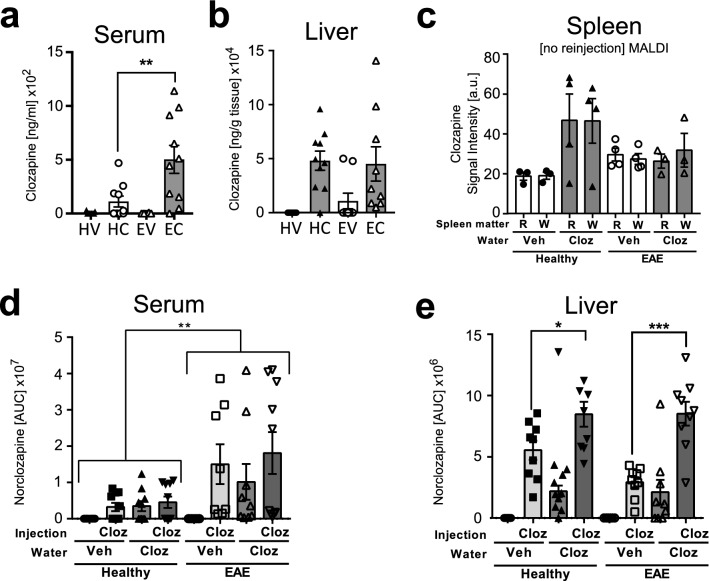
Clozapine and norclozapine quantification in the serum and liver and clozapine intensity in the spleen by MALDI**. (a,b)** Mice were treated with clozapine or vehicle 1 day prior to EAE induction in the drinking water and absolute clozapine concentration measured by LC–MS was analysed in the serum **(a)** and liver **(b)** (n = 10 mice/group), two-way ANOVA repeated measure followed by Sidak’s multiple comparison test, ** p > 0.01; **(c-e)** Mice were treated with clozapine or vehicle 1 day prior to EAE induction in the drinking water and clozapine signal intensity by MALDI IMS was analysed in the spleen, separated by red and white matter (n = 4–5 mice/group) **(c)** and norclozapine concentration measured by LC–MS was analysed in the serum **(d)** and liver **(e)** (n = 10 mice/group) in healthy and EAE mice treated with clozapine in the drinking water and reinjection of clozapine, two-way ANOVA repeated measure followed by Sidak’s multiple comparison test, *p > 0.05; **p > 0.01; ***p > 0.001.

To determine if clozapine levels in peripheral organs were altered by either chronic clozapine exposure or on-going neuroinflammation, we evaluated clozapine levels in the serum, spleen, liver, and lung. The liver is the major site for metabolism of clozapine into norclozapine and other metabolites; furthermore, given the reported immunomodulatory activity of clozapine and its effects in the EAE model, we chose to measure clozapine levels in the spleen, the major secondary lymphoid organ. Continuous treatment with clozapine in the drinking water of mice with EAE showed significantly higher levels of the drug in the serum compared to healthy animals (500 vs 100 ng/mL, EAE vs healthy; Fig. 3a; **p > 0.01; HV-healthy vehicle, HC-healthy clozapine, EV-EAE vehicle, EC-EAE clozapine). This effect was lost with re-injection (Fig. S2a). Absolute quantification of clozapine by LC–MS in the liver and lung and MALDI IMS analysis of the splenic red and white pulp showed no difference between healthy and EAE mice (Fig. 3b,c, HV-healthy vehicle, HC-healthy clozapine, EV-EAE vehicle, EC-EAE clozapine, R-red, W-white S2b to d). Together these results indicate that the distribution of clozapine in serum but not other tissues is altered by EAE.

Because the higher serum levels could reflect changes in clozapine’s metabolism, we measured the primary metabolite of clozapine, norclozapine (m/z 312.1142). No significant differences between groups was seen in the brain or the lung (Fig. S2e,f), but interestingly, a significant difference between healthy and EAE animals was detected in the serum, where significantly higher norclozapine levels were detected during EAE after chronic or single exposure to clozapine (Fig. 3d **p > 0.01). Furthermore, although no difference between healthy and EAE animals was observed in the liver samples, significantly higher levels of norclozapine were detected when clozapine was chronically administered before the single clozapine injection (Fig. 3e *p > 0.05; ***p > 0.001). Even though, hypermetabolism and changes in the metabolism of clozapine into other metabolites, like clozapine-*N*-oxide, cannot be ruled out completely, these findings indicate that the higher levels of clozapine in the serum during EAE were not due to impaired metabolism of clozapine to norclozapine in the liver.

### Clozapine does not alter dopamine levels or its localisation

**Figure 4:**
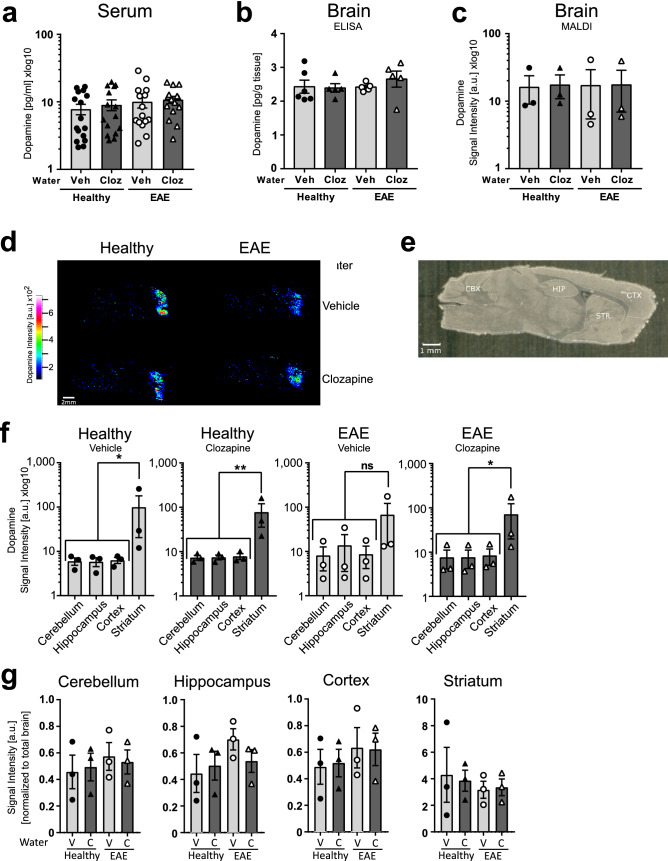
Dopamine quantification in the serum and brain. Mice were treated with clozapine or vehicle 1 day prior to EAE induction in the drinking water and different organs were collected and analysed for absolute concentration of dopamine in the serum (n = 15 mice/group) **(a)** or brain (n = 5 mice/group) **(b)** by ELISA. **(c)** Quantification of dopamine intensity by MALDI IMS in the whole mouse brain (n = 3 mice/group) **(d)** Representative MALDI images from all four treatment groups **(e)** Optical image for the segmentation into different brain regions. *CBX* cerebellum; *CTX* cortex; *HIP* hippocampus; *STR* striatum **(f)** clozapine intensity in the different brain region measured by MALDI separated by treatment group. (n = 3 mice/group) RM-one way ANOVA comparing Striatum to the others regions, *p > 0.05; **p > 0.01 **(g)** Clozapine intensity by different treatment in the different brain region measured by MALDI separated by brain regions. *C* clozapine; *V* Vehicle (n = 3 mice/group).

One of clozapine’s mechanisms of action is through antagonism of serotonin and dopamine receptors, which could change in vivo dopamine levels. The contribution of targeting the serotonin receptors, 5-HT2A/2C by clozapine is unclear, but there is evidence that it might have an effect on forebrain norepinephrine and dopamine neurotransmission^21^, which could change in vivo dopamine levels. Therefore, we analysed the concentration of dopamine in the serum and brain of healthy and EAE mice with or without clozapine treatment in the drinking water, because dopamine exerts its main effect as a hormone and neurotransmitter in the brain. Interestingly, we found that serum levels were similar between all groups (Fig. 4a) as were the levels in whole brain lysates (Fig. 4b). Since this analysis was done on the whole brain, we analysed brains by MALDI IMS to determine if clozapine treatment altered dopamine localisation (Fig. 4c–g). In all animals, dopamine intensity was highest in the striatum compared to the hippocampus, cortex or cerebellum while looking at each treatment individually (Fig. 4f; *p > 0.05; **p > 0.01). Showing the same data from Fig. 4f but sorted by the different brain regions, we could see that this distribution did not change with clozapine treatment or during EAE (Fig. 4g V-Vehicle, C- Clozapine) suggesting that clozapine does not significantly alter dopamine levels or distribution in the serum or CNS.

### EAE altered the expression of dopamine receptors on immune cells in the CNS

Since antagonism of dopamine receptors (DR) by clozapine^29^ has immunomodulatory effects^30–32^, the expression of all five DRs was analysed on brain-resident microglia and brain-infiltrating immune cells (CD4^+^T cells, CD11b^+^Ly6C^+^ monocytes and macrophages, Fig. S3). While CD4 + T cells play a key role in EAE and MS,^9^ monocytes and macrophages have also been shown to be important in EAE and MS^5, 33–36^ and to express dopamine receptors^21^. Moreover, clozapine has been reported to impair monocyte infiltration into the brain and spinal cord^6^. Thus, we included these myeloid cell types in the analysis of dopamine receptor expression in the CNS.

**Figure 5:**
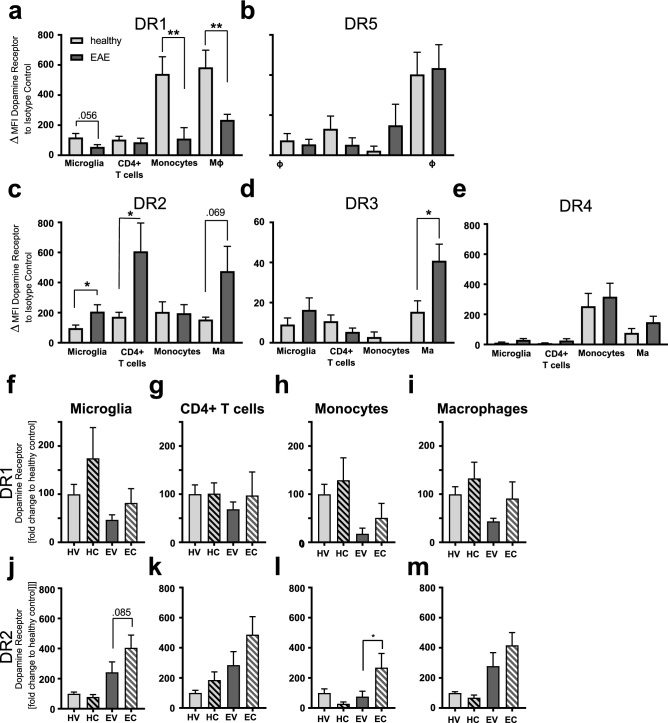
Dopamine receptor expression in the brain. Dopamine receptor expression on brain-resident or infiltrating immune cells in healthy mice compared to EAE mice **(a)** DR1 **(b)** DR5 **(c)** DR2 **(d)** DR3 **(e)** DR4 (n = 15 mice/group) Ordinary one-way ANOVA followed by sidak’s multiple comparison test comparing each cell type, * p > 0.05**,** ** p > 0.001. Mice were treated with clozapine or vehicle 1 day prior to EAE induction in the drinking water and immune cells were analysed for expression of dopamine receptors by flow cytometry. **(f)** DR1 expression on microglia **(g)** DR1 expression on CD4 + T cells **(h)** DR1 expression on monocytes **(i)** DR1 expression on macrophages **(j)** DR2 expression on microglia **(k)** DR2 expression on CD4 + T cells **(l)** DR2 expression on monocytes **(m)** DR2 expression on macrophages. (n = 10–15 mice/group) *HV* healthy vehicle; *HC* healthy clozapine; *EV* EAE vehicle; *EC* EAE clozapine, two-way ANOVA followed by Sidak’s multiple comparison test, * p > 0.05.

In healthy mice, DR1 expression (Fig. 5a) was most highly expressed on macrophages (ordinary one-way ANOVA followed by Tukey’s multiple comparison test comparing: microglia vs -macrophage p = 0.002; CD4 T cells vs -macrophage p = 0.005) and monocytes (ordinary one-way ANOVA followed by Tukey’s multiple comparison test: microglia- vs monocytes p = 0.007, CD4 T cells vs -monocytes p = 0.01) while DR5 expression was highest on macrophages (ordinary one-way ANOVA followed by Tukey’s multiple comparison test: microglia vs -macrophage p = 0.003; CD4 T cells vs -macrophage p = 0.04; monocytes vs -macrophage p = 0.002) and DR4 on monocytes (ordinary one-way ANOVA followed by Tukey’s multiple comparison test: microglia vs -monocytes p = 0.0002; CD4 T cells vs -monocytes p = 0.0005; monocytes vs -macrophages p = 0.008) (Fig. 5b,e). DR3 expression was low and DR2 similar on all cell types from healthy animals (Fig. 5c,d).

Interestingly, EAE led to a significant reduction in DR1 on macrophages and monocytes, but the reduction on microglia did not reach significance (Fig. 5a, ordinary one-way ANOVA followed by Sidak’s multiple comparison test comparing healthy vs EAE for each cell type (for all Fig. 5a–e). DR2 expression was significantly increased on microglia and CD4^+^T cells by EAE while the increase on macrophages did not reach significance (Fig. 5c). Although DR3 expression was low in healthy mice, EAE increased the expression on macrophages significantly (Fig. 5d). No significant EAE-induced changes were detected for DR4 or DR5 (Fig. 5b,e).

Analysing now the same data from the healthy and EAE DR expression analysis in more detail and taking the treatment with clozapine into account, we could see that overall, clozapine treatment significantly enhanced DR1 and DR2 expression on microglia, monocytes and macrophages even during EAE (p < 0.05 and p < 0.01, respectively Fig. 5f–m HV-healthy vehicle, HC-healthy clozapine, EV-EAE vehicle, EC-EAE clozapine, two-way ANOVA followed by Sidak’s multiple comparison test (for all Fig. 5m–f). Whereas no consistent clozapine-mediated effects on DR3, DR4 and DR5 expression was detected (Fig. S2g). These results indicate that EAE reduces the expression of DR1 and enhances the expression of type 2 DR (DR2 and DR3) on immune cells in the brain while clozapine upregulates both DR1 and DR2.

## Discussion

MS is characterised by demyelination of neuronal axons targeted by infiltrating autoreactive CD4^+^T cells. Using the EAE model to study neuroinflammatory MS, we have shown previously that clozapine effectively reduces disease severity^4^ and that this beneficial effect is not due to altered peripheral responses^5, 25^. Specifically, while clozapine does not inhibit antigen-specific Th1 or Th17 responses in the periphery, it modestly alters other peripheral T cell responses during EAE and promotes in vitro differentiation into Tregs^25^. However, these changes to peripheral T cells are not responsible for clozapine’s beneficial effects during EAE^25^. To determine whether protection was due to a direct action of clozapine in the CNS, we assessed the distribution and concentration of clozapine in the brain and other tissues of healthy and EAE animals by MALDI IMS and LC–MS. We found that although EAE did not alter the localisation or distribution of clozapine in the brain, liver lung, or spleen, it significantly increased the levels in the serum. Additionally, while dopamine levels were not affected by EAE or by clozapine, dopamine receptor (DR) expression on brain-isolated immune cells was significantly and differentially altered by EAE and clozapine suggesting that clozapine may act differently during EAE due to an altered expression of its target receptor. This study is the first in-depth report assessing clozapine, dopamine and dopamine receptors in the brain during EAE and after chronic clozapine administration and highlights how EAE-associated neuroinflammation may alter clozapine’s target pathways.

The finding that clozapine levels were significantly higher in the serum of EAE compared to healthy mice is intriguing because there were no differences detected in any other tissue. The higher levels were not due to impaired metabolism as norclozapine concentrations were also significantly elevated during EAE. Instead, it is possible that clozapine and norclozapine are retained in the serum, and previous research has shown that clozapine and its metabolites are strongly bound to the serum protein, α-1-acid glycoprotein^37^, with the free fraction of clozapine being less than 10%^38^. Additionally, higher concentrations of α-1-acid glycoprotein have been reported in the serum and plasma of MS patients with different stages of disease^39, 40^. Furthermore, a membrane bound α-1-acid glycoprotein can be synthesized by leucocytes and subsequently cleaved and released as the soluble serum form^41^. Because, leucocytes are activated in EAE, it is possible that immune cells are responsible for an increase in α-1-acid glycoprotein, which binds and retains clozapine in the blood. Peak serum levels of clozapine are reached 1–6 h after oral dosing and the half-life is about 4 to 16 h in healthy adults^42^, and our findings suggest this half-life may be extended during a neuroinflammatory response.

MALDI IMS was used for the first time to analyse the distribution of unlabelled clozapine within the brain tissue from healthy and EAE mice. Previously, Quiason and Shahidi-Latham used a DHB matrix on brain samples from rats treated orally with clozapine and reported a good detection of the drug in the brain tissue^23^. We also found DHB gave the best results compared to other matrices (data not shown); however, in our study, the oral-administered clozapine dose was too low to detect consistently in the brain. Therefore, we injected 10 mg/kg clozapine 30 min before euthanasia and were able to detect clozapine reproducibly. Similarly, Hsieh et al. showed by MALDI IMS that intravenous injections of clozapine into rats resulted in a high detection of clozapine in the brain after 45 min^43^ and Baldessarini et al. showed by reversed-phase liquid chromatographic separation, that detection levels peak at 30 min after 10 mg/kg intraperitoneal injection^26^.

With the segmentation of the brain into six different brain regions, we showed that clozapine localises to specific brain regions, with highest intensity in the cortex and thalamus and lowest in the cerebellum and medulla. Using desorption electrospray ionization IMS, clozapine was also detected in healthy rat brains after oral dose of 50 mg/kg clozapine, with the highest levels in the cortical regions^38^. Brain PET scan imaging from healthy humans injected with^11^C-labelled clozapine showed that clozapine seems to localize to specific brain regions with the highest intensity in the insula and striatum and the lowest in the cerebellum^27^, and our results are consistent with these findings.

The high concentrations in the liver compared to other organs are most likely because the liver is the major site where clozapine is metabolized. The cytochrome P450 system, in particular CYP1A2, converts clozapine into the two main primary metabolites norclozapine and clozapine-*N*-oxide^44^. Experiments in CYP1A2-null mice showed a significant longer half-life in the serum and a reduced time of clearance^45^. This finding likely explains the high clozapine concentrations in the liver after acute exposure. Over time, clozapine concentrations reduce due to its metabolism and only low levels can be detected in the continuously treated animals. While an ion suitable for clozapine-*N*-oxide was detected in some liver samples, it’s presence and concentration were inconsistent. Interestingly, we did see increased norclozapine intensity in liver samples from mice continuously treated with clozapine in the drinking water. This leads to the conclusion that clozapine metabolites accumulate in the liver over time and have a longer half-life.

One mechanism of clozapine’s action is through antagonism of serotonin and dopamine receptors (DR). Our choice to investigate dopamine was based upon clozapine’s known targets and the involvement of those targets in EAE. Clozapine was developed to antagonize DR2 but has greater affinity for DR1, serotonin receptors (esp 5-HT2A and C subclasses), alpha adrenergic receptors, and histamine receptor 1^46^. At the time we began this study, previous research had shown that selective DR1 antagonism reduces EAE while selective DR2 antagonism exacerbates EAE indicating that regulation of the dopamine receptor pathways is important^16^. In contrast, serotonin reuptake inhibitors as well as a novel 5-HT1A/D2 ligand have been shown to ameliorate EAE suggesting a positive role for serotonin in MS^47–49^. These findings suggest that clozapine’s ability to reduce EAE is not principally through serotonin receptor antagonism although further research to assess the contribution of 5-HT2A and C is warranted, but that dopamine receptors look to be involved. Based upon these studies, we focused our investigation on dopamine and not serotonin.

The specific role of dopamine and its receptors is still unclear but several previous studies have shown that the changes in dopamine might be related to inflammation in autoimmune diseases^50^. We have shown here that dopamine levels do not change in the serum of healthy or EAE mice despite continuous clozapine treatment in the drinking water. In addition, dopamine levels in the brain tissue measured by ELISA or by MALDI IMS were not different. However, we identified the striatum as the major site of dopamine localisation and it did not change in any condition tested. This finding agrees with several studies where high dopamine levels were detected in the striatum by HPLC^51–53^. Although Balkowiec-Iskra et al. reported higher concentrations than our study, these reports differ in tissue (isolated striatum vs whole brain) and method (HPLC vs MALDI IMS)^44^. Increased dopamine levels after clozapine injection were previously reported in the rat caudate, nucleus accumbens and the medial prefrontal cortex measured by HPLC^46–48^, which is a more sensitive method compared to MALDI IMS. Similarly, these studies assessed specific brain regions compared to our analysis in whole brains, which would dilute the dopamine levels. In addition, most of these studies injected clozapine i.p. and studied the dopamine concentration within one hour after injection while our treatment regime was in a continuous treatment of clozapine in the drinking water. Meltzer et al. have shown that dopamine levels peak 60 min after clozapine injection and then return to baseline levels^46^. This immediate and high response of dopamine to the clozapine injection may differ from the continuous lower levels of clozapine achieved by administration in the drinking water because it might depend on when the mice were last drinking. Future studies investigating the effect of chronic, low dose clozapine on dopamine, may need to assess not only dopamine but also its metabolites like DOPAC^47^.

Interestingly, the regions with the highest clozapine levels are known to express a high density of DR1 and DR2, and the cerebellum, with low dopamine levels, has lower DR expression^54^. All DRs are reported to be widely expressed in the CNS and several studies have reported their expression on immune cells^11^, which play an important role in MS^50^. Moreover, DR expression on immune cells is also associated with some neurological and immune-related disorders^55, 56^. But, to our knowledge, this study is the first to monitor DRs on brain-resident cells and infiltrating immune cells during EAE. Here, we show that all five DR are present on brain-resident microglia, although the levels were lower than on other myeloid cells (i.e. monocytes, and macrophages). Furthermore, we found that EAE upregulated D2 subtypes (DR2 and DR3) and downregulated DR1. Interestingly, clozapine had an enhancing effect on DR1 and DR2 expression on cells from healthy and EAE animals. This study does not exclude any beneficial effects of clozapine on peripheral immune cells although our previous studies have shown that clozapine does not directly alter peripheral CD4^+^ T cells during EAE^25^. Instead, we have reported that clozapine modifies peripheral and CNS-resident innate immune cells with the greatest effects found in the CNS^6^. Therefore, this study has focused on DR expression on infiltrating and CNS-resident cells. Given the fascinating actions of dopamine outside of the CNS, further work needs to be performed to investigate DR expression on immune cells in the periphery and CNS at different stages of EAE, given that DR expression on immune cells is highly dynamic, context-sensitive and variable^57^.

A solid body of evidence indicates that in MS, dopaminergic pathways are dysregulated in the peripheral immune system. In general, human T cells express all DRs on their surface^57^, but the level of receptor expression differs between different T cell populations and activation states, and between resting and activated T cells. In healthy donors, resting T cells showed only a minimal expression of DR2-5 and no DR1 expression^58^. In line with our data, D1 subtypes are decreased on effector T cells from MS patients, and dopamine does not affect these cells^57^. Additionally, decreased DR5 expression could be detected, and DR5 performs an inhibitory role on T cells during MS^56^. Besides this evidence that DR are modulated during MS, dopamine showed only very limited therapeutic effects^50^.

Overall, this study is the first to show that clozapine localises to specific brain regions, which are known for high expression of the DR. While the localisation of clozapine was not altered by EAE, we found that clozapine levels were significantly elevated in the serum along with its metabolite norclozapine. Finally, immune cells in the CNS during EAE had decreased levels of DR1 but increased DR2, both of which were enhanced by clozapine administration. Overall, these findings indicate that clozapine may be retained for longer during neuroinflammation and mediate differential effects on immune cells through its target receptors, and together suggest that clozapine administration may have unexpected actions during MS due to modification of dopamine pathways, which is in line with a recent clinical trial assessing clozapine administration during MS^8^.

## Material and methods

All methods were carried out in accordance with relevant guidelines and regulations.

### Animals

Eight-ten-week old female C57BL/6J mice were purchased from the Biomedical Research Unit of the Malaghan Institute of Medical Research (Wellington, NZ) and housed in the Victoria University of Wellington PC2 animal facility at a 12 h dark–light cycle with food and water ad libitum.

### Ethics statement

All animal experiments were carried out in the School of Biological Sciences Animal Facility at Victoria University of Wellington and were approved by the Victoria University of Wellington Animal Ethics Committee (2014-R23 and 25295). All methods were carried out in accordance with relevant guidelines and regulations and in compliance with the ARRIVE guidelines.

### EAE induction and treatments

Mice were immunized s.c. in the rear flanks with myelin oligodendrocyte glycoprotein (MOG)_35–55_ peptide (50 µg/mouse; Genescript, Piscataway, NJ USA) in complete Freund’s adjuvant (Sigma, St Louis, MO USA) containing 500 µg/mouse *Mycobacterium tuberculosis* (Fort Richard, Auckland, NZ). In addition, mice were injected i.p. with pertussis toxin (200 ng/mouse; List Biochemicals, Campbell, CA USA) on days 0 and 2. Mice were weighed and scored daily as follows: 0, normal; 1, partial tail paralysis; 2, full tail paralysis; 3, paralysis in one hind limb; 4, paralysis in both hind limbs; and 5, moribund. One day before immunization drinking water was changed to 60 mg/kg/day clozapine (Douglas Pharmaceuticals Ltd., Auckland, NZ) or vehicle (0.1 M acetic acid). Mice were injected i.p. with 10 mg/kg clozapine or vehicle 30 min prior to CO_2_ euthanasia. Spleen, liver, lung and brain were collected and immediately snap-frozen in liquid nitrogen. Serum was collected as described previously^6^. Organs and serum were stored at − 80 °C until analysis.

### Tissue preparation for HPLC–MS analysis

Liver (70 mg) and lung tissue (20 mg) were homogenized in 1 mL 0.1 M acetic acid using a Polytron tissue homogenizer. Solutions were resuspended and centrifuged for 5 min at 1000 g, supernatant removed and centrifuged a second time (5 min at 14,000×*g*). Brain sections 10 µm thick) were cut on a Leica CM3050 cryotome (Leica Microsystem, Wetzlar, Germany). Five sections/brain were homogenized in 100 µL 0.1 M acetic acid. Solutions were resuspended using a pipette, centrifuged for 5 min at 1000 g and supernatant was spin filtered through a 30 K Microcon Centrifugal Filter (Millipore, Burlington, MA USA). Serum samples were mixed 1:1 with acetonitrile, vortexed 30 s and centrifuged 5 min at 14,000×*g*. All sample were transferred to vials containing a glass insert for small volumes (ThermoFisher, Waltham, MA USA). For the clozapine standard curves, clozapine was dissolved in 0.1 M acetic acid and standard curves were spiked in untreated healthy serum or tissue samples before sample preparation. All samples and standards were spiked with quetiapine (m/z 384.174, Douglas Pharmaceuticals Ltd.) as an internal standard.

### QTOF LC–MS analysis

An Agilent 1260 HPLC with a refrigerated autosampler (4 °C) equipped with a Synergi Hydro-RP column (50 × 2.0 mm, 2.5 µm, 100 Å, Phenomenex, Torrance, CA USA; Mobile phase in Table S1) was used for chromatography and samples were kept at 4 °C during the time of measurement with column temperature at 35 °C. An Agilent 6530 Accurate Mass Q-TOF LC–MS system (Santa Clara, CA USA) was used for detection with the settings shown in Table S1. Two scans per second were acquired between m/z 100 and 3200 with constant infusion of reference ions to maintain mass accuracy.

### Tissue preparation for MALDI IMS

Brain samples were sectioned as above, thaw mounted onto MALDI target plates or indium tin oxide-coated slides and stored at − 80 °C until analysis. Sections for dopamine detection were collected approximately 1 mm away from the midline.

### MALDI matrix application for clozapine detection

Tissue sections were dried in a vacuum desiccator for 30 min prior to scanning on a flatbed scanner (Epson Expression 10,000 XL; Suwa, Nagano, Japan) and 0.5 µl calibration mix was added outside of the tissue (containing clozapine, angiotensin II, bradykinin fragment 1–7, P14R and adrenocorticotropic hormone fragment 18–39). A TM-sprayer (HTX Imaging, Chapel Hill, NC USA) was used to spray-coat the sections with the DHB matrix as specified in Table S2. Every second pass the nozzle was rotated 90° to ensure equal distribution of the matrix. The last two passes had a track offset of 1.5 mm.

### MALDI matrix application and derivatisation for dopamine detection

TPP-TFB derivatisation matrix was sprayed on the tissue with the TM-sprayer as specified in Table S2. The tissue sections were incubated at RT for 2 h, then spray-coated with detection matrix CHCA as specified in Table S2 and incubated an additional 2 h at RT. The final concentrations of TPP-TFB and CHCA were 47 ng/mm^2^ and 2.5 µg/mm^2^, respectively.

### MALDI imaging mass spectrometry (IMS)

The spray-coated sections were analysed with an AB Sciex TOF/TOF 5800 mass spectrometer (Framingham, MA USA) operated as specified in Table S3. The laser intensity was optimized for each sample set. The mass spectrometer was externally calibrated using calibration peptides and clozapine as reference masses. The imaging resolution was set to 50 µm for clozapine and 100 µm for dopamine. The imaging data was stored in the Analyze 7.5 format.

### Data processing

MALDI IMS data was processed with msIQuant software^59^. Prior to loading the files into msIQuant, the data files were converted from the Analyze 7.5 format to the imzml format using the converter module from Spectviewer^60^, kindly provided by Jean-Pierre Both (French Atomic Energy Commission, France). Pre-processing of all images included background correction and spectral alignment. Image normalisation was based on the root mean square in msIQuant. Tissue sections used for clozapine detection were segmented into different brain regions based on three ion signals (m/z 488.01, 848.65 and 872.61), while tissue sections used for dopamine detection were segmented based on the optical image.

QTOF LC–MS data was analysed using the Qualitative Analysis B.06.00 from Agilent.

### Dopamine ELISA detection in serum and brain

Serum was collected and used immediately. For the brain, one brain hemisphere was mashed in PBS (0.1 mL PBS per 100 mg tissue). Samples were stored at – 20 °C. Two freeze and thaw cycles were performed, lysates were centrifuged at 5000×*g* for 5 min at 4 °C and supernatants were assayed immediately. Dopamine was measured using mouse dopamine ELISA kit from CUSABIO (CSB-E08661m; Houston, TX USA) according to manufactures instructions^61^. Plates were read at 450 nm using a PerkinElmer (Waltham, MA USA) microplate reader.

### Flow cytometry

Each brain was processed into a single-cell suspension and stained as described previously^6^ (Supplementary Table S4), and each sample of isolated cells was split into 5 parts for detection of the different dopamine receptors (Panel design Supplementary Table S5)^62–64^. After incubation with the primary antibodies, cells were incubated with the secondary antibody, FITC goat anti-rabbit IgG (BD Biosciences, San Jose, CA USA) or isotype control for 25 min. Flow cytometry was performed on a BD FACS Canto II (BD Biosciences) and analysed using FlowJo software version 10.1 (Treestar Inc, Woodburn, OR USA).

### Statistical analyses

All graphs and statistical analyses were generated using GraphPad Prism 7 (GraphPad Software Inc., San Diego, CA USA). For comparison of more than two groups one-way or two-way analysis of variance (ANOVA) was used with the recommended multiple comparison tests as indicated in the figure legend and as recommended by GraphPad Prism.

## Supplementary Information

Supplementary Information.

## Data Availability

The datasets used and/or analysed during the current study are available from the corresponding author on reasonable request.

## Abbreviations

ANOVA: Analysis of variance
cAMP: Cyclic adenosine monophosphate
CD4: Cluster of differentiation 4
DHB: 2,5-Dihydroxybenzoic acid
DR: Dopamine receptors
EAE: Experimental autoimmune encephalomyelitis
HPLC: High performance liquid chromatography
IMS: Imaging mass spectrometry
LC–MS: Liquid chromatography coupled mass spectrometry
MALDI: Matrix-assisted laser desorption ionization
MOG: Myelin oligodendrocyte glycoprotein
MS: Mass spectrometry
PET: Positron emission tomography
PBS: Phosphate-buffered saline
QTOF: Quadrupole time-of-flight mass spectrometry
RRID: Research resource identifier (see scicrunch.org)
