# Supplementary material for: Localisation of clozapine during experimental autoimmune encephalomyelitis and its impact on dopamine and its receptors
Source: Sci Rep. 2021 Feb 3;11:2966. doi: 10.1038/s41598-021-82667-6 (PMC7858600; doi:10.1038/s41598-021-82667-6)
Supplement: Supplementary file 1 — Supplementary Information. [file 41598_2021_82667_MOESM1_ESM.pdf]

Localisation of clozapine during neuroinflammation and its impact on dopamine and its receptors.

Katharina Robichon, PhD [1,2]*, Sven Sondhauss [1,2]*, T. William Jordan, PhD [1,2], Robert A. Keyzers, PhD [2,3], Bronwen Connor, Prof. [4], Anne C. La Flamme, Prof. [1,2,5]

[1] School of Biological Sciences, Victoria University of Wellington, Wellington, New Zealand

[2] Centre for Biodiscovery Wellington Victoria University of Wellington, Wellington, New Zealand

[3] School of Chemical and Physical Sciences, Victoria University of Wellington, Wellington 6140, New Zealand

[4] Department of Pharmacology and Clinical Pharmacology, Centre for Brain Research, University of Auckland, Auckland, New Zealand

[5] Malaghan Institute of Medical Research, Wellington, New Zealand

* These authors contributed equally

Corresponding author: Prof Anne Camille La Flamme, School of Biological Sciences, Victoria University of Wellington, P.O. Box 600, Wellington, 6140 New Zealand +64-4-463-6093 (office) anne.laflamme@vuw.ac.nz (e-mail)

Running Title: Clozapine localisation in healthy and EAE mice

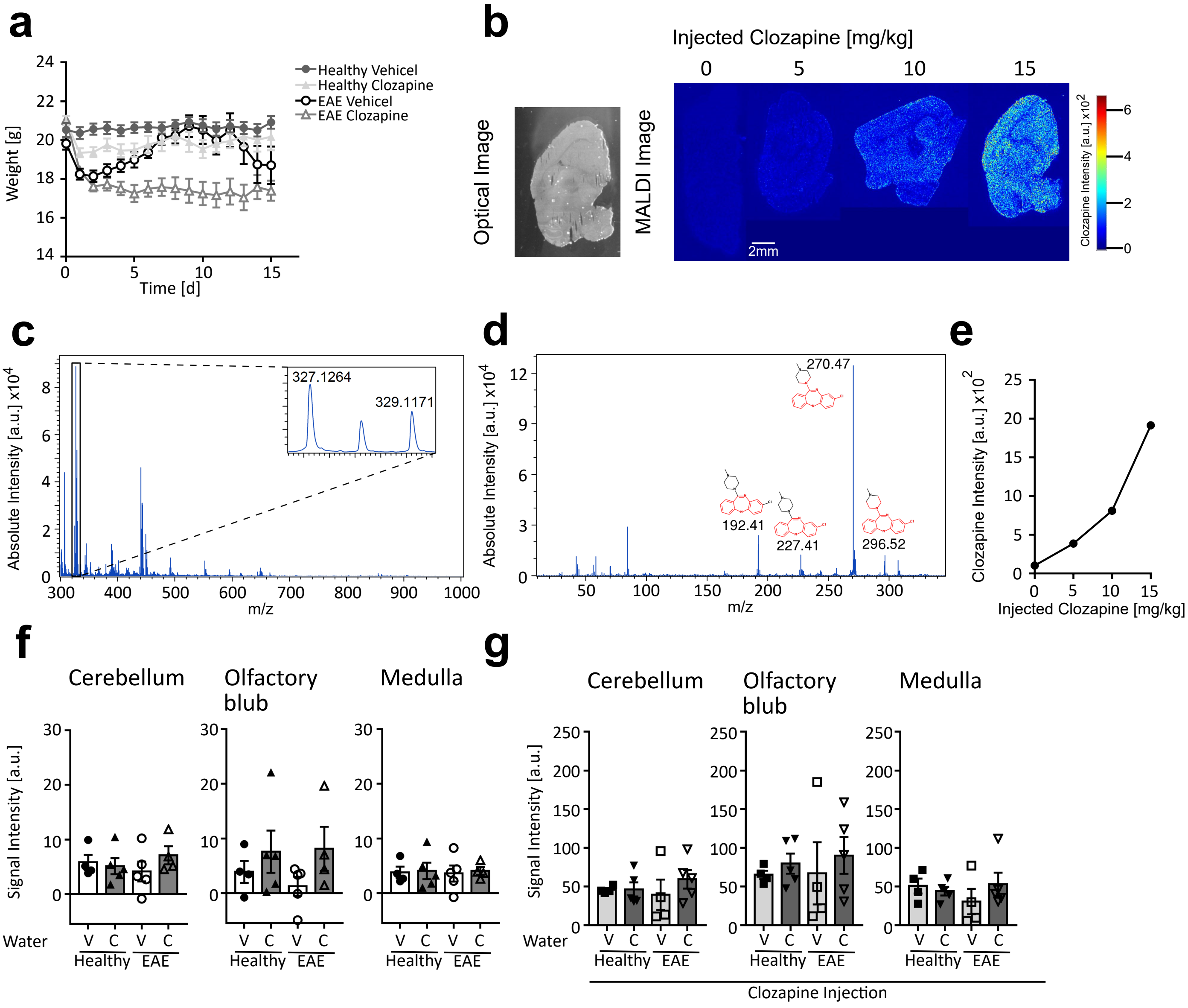

Supplement Fig. 1 Clozapine optimization of detection in mouse brain and Clozapine intensity in different brain regions. Mice were treated with clozapine or vehicle 1 day prior to EAE induction in the drinking water and scored daily (0: normal to 5: moribund). (a) Weight of mice. Shown are the means and SEM of individual mice from two experiments combined (n = 20 mice/group). Optical Image of mouse brain and MALDI images of clozapine dose response in the mouse brain (b). MALDI mass spectrum of clozapine with DHB as matrix; small box: zoom in on clozapine peaks with characteristic isotopic distribution (c) Fragmentation mass spectrum of clozapine (parent ion m/z 327.12). The respective fragment is highlighted in the red in the chemical structure of clozapine (d). Quantification of clozapine intensity in the mouse brain after increasing i.p injection (n = 2) (e). (f) Clozapine intensity in the different brain region measured by MALDI in mice with clozapine in the drinking water (n = 4-5 mice/group). (g) Clozapine intensity in cerebellum, olfactory bulb and medulla in healthy and EAE mice after clozapine re-injection C – clozapine; V - vehicle.

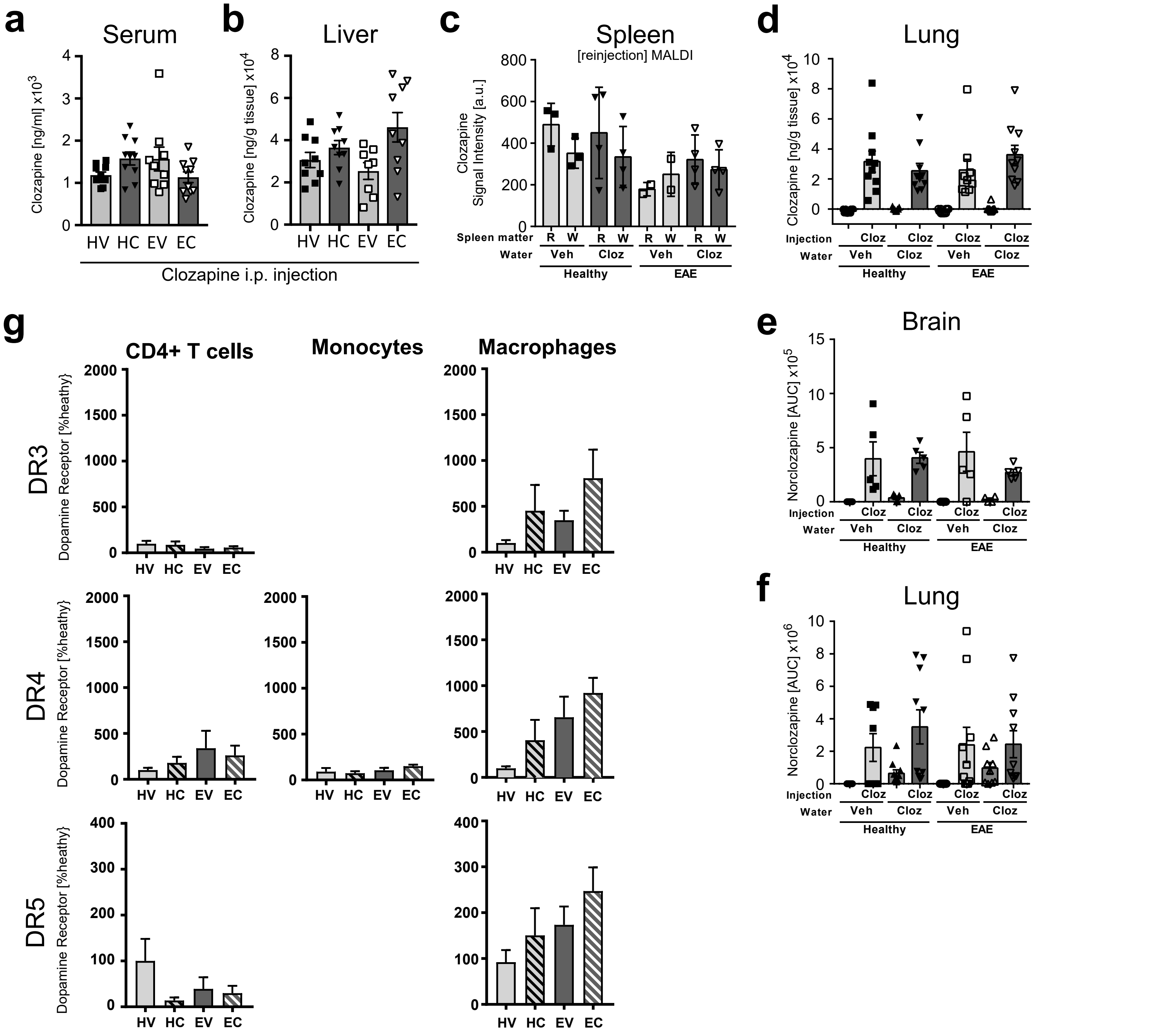

Supplement Fig. 2 Clozapine and norclozapine quantification in the serum, liver and lung, clozapine intensity in the spleen by MALDI and dopamine receptor expression in the brain. Mice were treated with clozapine or vehicle 1 day prior to EAE induction in the drinking water and injected i.p. with 10 mg/kg clozapine 30 minutes before euthanization. Different organs were collected and absolute clozapine concentration measured by LC/MS was analysed in the serum (a), liver (b) and lung (d) (n = 10 mice/group) and clozapine signal intensity by MALDI IMS was analysed in the spleen, separated by red and white matter (n = 4-5 mice/group)(C). Absolute norclozapine concentration measured by LC/MS was analysed in the brain (e) and lung (f) (n = 10 mice/group). (g) Mice were treated with clozapine or vehicle 1 day prior to EAE induction in the drinking waterand immune cells were analysed for expression of dopamine receptors by flow cytometry. DR3 expression on CD4+ Tcells, monocytes and macrophages, DR4 expression on monocytes, DR5 expression on CD4+ Tcells, monocytes and macrophages. (n = 10-15 mice/ group) HV – healthy vehicle; HC – healthy clozapine; EV – EAE vehicle; EC – EAE clozapine

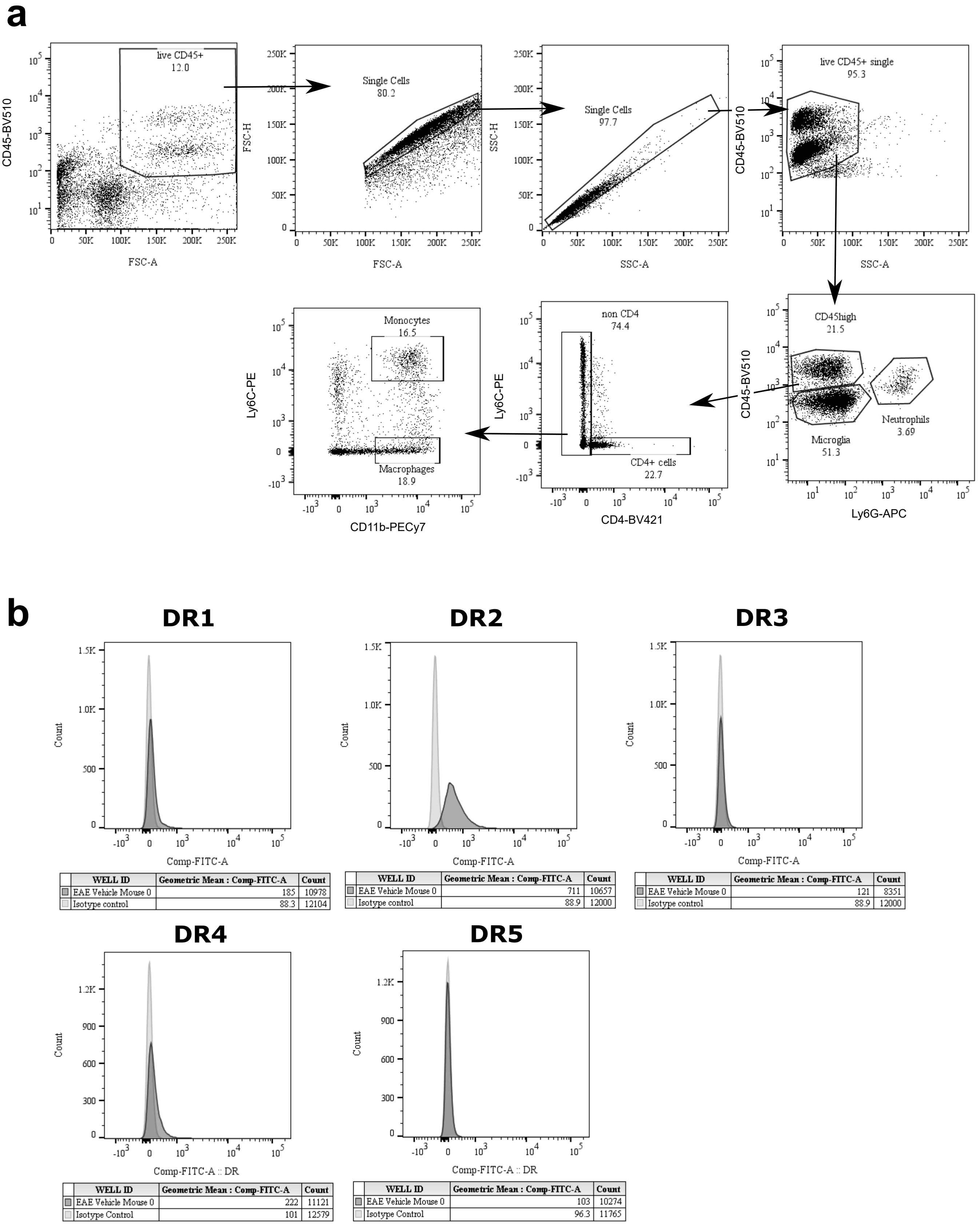

Supplement Fig. 3 Gating strategy for dopamine receptor expression by flow cytometry. Gating strategy is shown for the (a) the different cell populations in the brain and (b) for all 5 dopamine receptor expressions on microglia from one EAE vehicle treated animal as an example, the following flow cytometry markers are used: CD4-BV521 (RM4-5), CD45-BV510 (30-F11), CD8-PerCPCy5.5 (53-6.7), CD11b-PE-Cy7 (M1/70), CD3-APC-Cy7 (17A2), Ly6C-PE (HK1.4), Ly6G-APC (1A8) and dopamine receptor secondary Antibody FITC Goat Anti-Rabbit IgG

Table S1 HPLC-Mass Spectrometer specifications

**Mobile phase A**	5 mM ammonium formate and 0.1% formic acid in water	
**Mobile phase B**	Acetonitrile	
**Mobile phase gradient**	0 - 0:50 min	B 5%
	14 – 15 min	B 100%
	15:10 - 18 min	B 5%
**Mobile phase flow rate**	0.4 mL/min	
**Ionization**	Mode	Positive ESI
	Source	JetStream electrospray
	Drying gas temperature	275 °C
	Flow rate	9 L/min
	Nebuliser pressure	40 psi
**Sheath gas**	Flow	10 L/min
	Temperature	300 °C
**Electrospray capillary**	Voltage	4000 V
**Nozzle**	Voltage	500 V
**Fragmentor**	Voltage	100V

Table S2 MALDI matrix application with TM-sprayer specifications

**Clozapine detection**		
	Matrix	2,5-Dihydroxybenzoic acid (DHB) (Sigma, USA)
	Matrix solution	50% ACN, 0.2% trifluoroacetic acid
	Matrix concentration	35 mg/ml
	Number of passes	6
	Nozzle speed	600 mm/min
	Track spacing	3 mm
	Nozzle Temperature	80°C
	Flow rate	80 µL/min
**Dopamine detection**	**TPP-TFB**	
	Matrix	2,4,6-Triphenylpyrylium tetrafluoroborate (TPP-TFB, Sigma, USA)
	Matrix solution	Methanol, 0.5 % triethylamine
	Matrix concentration	0.15 mg/ml
	Number of passes	8
	Nozzle speed	600 mm/min
	Track spacing	3 mm
	Nozzle Temperature	50°C
	Flow rate	70 µL/min
	**CHCA**	
	Matrix	α-Cyano-4-hydroxycinnamic acid (CHCA, Merck)
	Matrix solution	70 % ACN, 0.2 % TFA
	Matrix concentration	8 mg/ml
	Number of passes	8
	Nozzle speed	600 mm/min
	Track spacing	3 mm
	Nozzle Temperature	75°C
	Flow rate	70 µL/min

Table S3 MALDI IMS specifications

**Reflector ion mode**	positive	
**Mass range**	m/z 240-1000	
**Bin size**	1ns	
**Voltage settings**	Source	15 kV
	Grid	11.55 kV
	Focus	7.5 kV
**Laser diameter**	just below 50 µm	

Table S4 Flow Cytometry Antibodies

Target	Flurophore	Clone	Company
CD4	BV421	RM4-5	Biolegend
CD45	BV510	30-F11	Biolegend
CD8	PerCP-Cy5.5	53-6.7	Biolegend
CD11b	PE-Cy7	M1/70	Biolegend
CD3	APC-Cy7	17A2	Biolegend
Ly6C	PE	HK1.4	Biolegend
Ly6G	APC	1A8	Biolegend
DR1		ab20066	Abcam
DR2		324393	Millipore
DR3		ab42114	Abcam
DR4		ab135978	Abcam
DR5		ab181623	Abcam

Table S5 Flow Cytometry Panel design

Laser	Channel	Fluorophore	Panel 1	Panel 2	Panel 3	Panel 4	Panel 5
Violet (405)	Blue	BV421	CD4	CD4	CD4	CD4	CD4
	Green	BV510	CD45	CD45	CD45	CD45	CD45
Blue (488)	Green	FITC	**DR1**	**DR2**	**DR3**	**DR4**	**DR5**
	Yellow	PE	Ly6C	Ly6C	Ly6C	Ly6C	Ly6C
	Red	PerCP-Cy5.5	CD8	CD8	CD8	CD8	CD8
	Infrared	PE-Cy7	CD11b	CD11b	CD11b	CD11b	CD11b
Red (633)	Red	APC	Ly6G	Ly6G	Ly6G	Ly6G	Ly6G
	Infrared	APC-Cy7	CD3	CD3	CD3	CD3	CD3
